# The Impact of Concomitant Traumatic Brain Injuries on the Surgical Treatment of Burns: A Long-Term, Monocentric Retrospective Study

**DOI:** 10.1093/jbcr/iraf216

**Published:** 2025-11-24

**Authors:** Mauro Vasella, Michael-Alexander Pais, Lukas Naef, Matthias Haenggi, Giovanna Brandi, Emanuela Keller, Victor E Staartjes, Luca Regli, Pietro Giovanoli, Bong-Sung Kim, Flavio Vasella

**Affiliations:** Department of Plastic Surgery and Hand Surgery, University Hospital Zurich, 8091 Zurich, Switzerland; Department of Plastic Surgery and Hand Surgery, University Hospital Zurich, 8091 Zurich, Switzerland; Department of Plastic and Reconstructive Surgery, BG Trauma Center Ludwigshafen, University of Heidelberg, 67071 Ludwigshafen, Germany; Department of Plastic Surgery and Hand Surgery, University Hospital Zurich, 8091 Zurich, Switzerland; Institute of Intensive Care Medicine, University Hospital Zurich and University of Zurich, 8091 Zurich, Switzerland; Institute of Intensive Care Medicine, University Hospital Zurich and University of Zurich, 8091 Zurich, Switzerland; Institute of Intensive Care Medicine, University Hospital Zurich and University of Zurich, 8091 Zurich, Switzerland; Department of Neurosurgery, Clinical Neuroscience Center, University Hospital Zurich and University of Zurich, 8091 Zurich, Switzerland; Department of Neurosurgery, Clinical Neuroscience Center, University Hospital Zurich and University of Zurich, 8091 Zurich, Switzerland; Department of Plastic Surgery and Hand Surgery, University Hospital Zurich, 8091 Zurich, Switzerland; Department of Plastic Surgery and Hand Surgery, University Hospital Zurich, 8091 Zurich, Switzerland; Department of Neurosurgery, Clinical Neuroscience Center, University Hospital Zurich and University of Zurich, 8091 Zurich, Switzerland

**Keywords:** burns, traumatic brain injury, surgical timing, complications, multidisciplinary management

## Abstract

Burn injuries significantly impact morbidity and mortality, with early surgical intervention crucial for improving outcomes. However, concomitant traumatic brain injury (TBI) frequently complicates burn management, potentially delaying timely surgical treatment due to neurological concerns. Optimal timing of burn surgery in patients with concurrent TBI remains uncertain, necessitating clearer insights into their clinical outcomes. This retrospective study reviewed burned adults admitted to a Swiss Burn Center between 2014 and 2023. Patients were grouped as burns with TBI, burns alone, burns with other trauma, or electrical burns. Demographics, injury characteristics, timing of surgery, complications, and outcomes were analyzed. Generalized linear models and logistic regression were applied. Among 602 patients, 27 (4.5%) had a TBI. Mortality was highest in this group (22.2%) compared to isolated burns (3.7%), burns with other trauma (7.4%), and electrical burns (4.8%). Surgical delays (>72 h) occurred only in burn patients with TBI (22.2%), mainly due to hemodynamic instability, intracranial pressure monitoring, or additional trauma. Delayed surgery correlated with more surgical interventions (*P* = .018) and longer operative times (*P* = .048). Complications were more frequent: wound infections (48.1%) and graft loss (22.2%) were significantly higher in the TBI group. In conclusion, burns with concomitant TBI define a distinct, high-risk subgroup with increased surgical delays, complications, and mortality. Management requires an interdisciplinary approach, balancing early surgical intervention with neuroprotective strategies to optimize patient outcomes.

## INTRODUCTION

Burn injuries are among the most severe forms of trauma, leading to significant morbidity and mortality worldwide. Prompt surgical intervention, particularly early excision and grafting, is crucial in the management of burns to reduce infection risk, improve healing times, and enhance overall outcomes.^1^ However, the presence of concomitant injuries can complicate this timely surgical approach. Traumatic brain injury (TBI) is a common co-occurring injury in patients with burns, especially in settings involving explosions, high-velocity impacts, or falls.^2^^,^^3^

The management of patients with both burns and TBI presents unique challenges. The physiological stress from burns can exacerbate cerebral edema and intracranial pressure (ICP), while concerns about hemodynamic stability and the effects of anesthesia and volume management on intracranial dynamics may force clinicians to delay surgical interventions.^4^ Moreover, the need for neurological monitoring and the risk of secondary brain injury may further complicate the decision-making process regarding the timing of burn wound excision.^3^

Existing literature has explored various aspects of burn management and TBI separately, but there is a paucity of studies specifically addressing the impact of TBI on the timing of surgical interventions in burn patients.^5^^,^^6^ Some studies suggest that delays in surgical treatment of burns can lead to worse outcomes, including increased infection rates, prolonged hospital stays, and higher mortality.^1^^,^^7^ However, the balance between the risks of delayed burn surgery and the potential for TBI exacerbation remains unclear.

Understanding the interplay between TBI and burns is essential for optimizing patient care. Delays in surgical intervention due to TBI may adversely affect burn outcomes, yet early surgery may pose risks to neurological recovery. Given the limited evidence, a clearer understanding of how TBI affects surgical timing and patient outcomes is crucial.

This study aims to address this gap by retrospectively analyzing the timing of surgical interventions in patients with burns, comparing those without and with concomitant TBI. By examining admission records, surgical timing, and patient outcomes over a 10-year period, this research seeks to provide insights into the management of this complex patient population and contribute to the development of evidence-based guidelines for optimizing care.

## MATERIALS AND METHODS

### Study design

Approval for this retrospective study was obtained from the local ethical committee and registered in accordance with the Declaration of Helsinki in the “Registry of all Projects in Switzerland” of Swiss Ethics Committees on research involving humans (BASEC ID 2024-01978). Additionally, approval of general consent (GC) was obtained from the patients or their next of kin for data collection. Analyzed data included the years between 2014 and 2023 from patients admitted to the intensive care unit (ICU) of a Burn Center in Switzerland. Exclusion criteria were a rejected GC or patients under 16 years of age. In this study, 4 patient cohorts were defined based on injury type: burns with concomitant TBI, burns alone, burns combined with other trauma (excluding TBI), and electrical burn injuries. The patients selected for the non-TBI groups were adjusted for severity of burn injuries and matched as closely as possible for key baseline variables (age, gender, TBSA, abbreviated burn severity index [ABSI], burn depth), allowing a meaningful comparison of the groups. This approach helped ensure comparability and minimized baseline differences. Baseline characteristics, including demographic information, various burn etiologies, injury mechanisms, burn-specific features, treatment details, complications, discharge outcomes, and mortality data, were summarized using frequency distribution tables. To analyze the associations between these characteristics and predefined outcome measures, we applied generalized linear models (GLMs) for count data and logistic regression analyses for categorical variables, respectively.

### Patient demographics and comorbidities

A range of patient-related information, including age, gender, and obesity status, with obesity defined as a body mass index of 30 kg/m^2^ or higher, was gathered. The presence of diabetes mellitus, peripheral artery disease, coronary heart disease, heart failure, and chronic obstructive pulmonary disease (COPD) was also recorded. Histories of regular alcohol or nicotine use, pre-existing psychiatric conditions, multiple psychiatric diagnoses (2 or more), prior suicide attempts, use of controlled or illicit substances, and statuses of unemployment or retirement were also documented.

Pre-existing psychiatric conditions were identified based on the International Classification of Diseases, 10th Revision (ICD-10), or the Diagnostic and Statistical Manual of Mental Disorders, Fifth Edition (DSM-5).^8^

Controlled substances included general antipsychotics, tricyclic antidepressants, sedatives, or their combinations. The controlled substances examined were tetrahydrocannabinol, methadone, buprenorphine, other cannabinoids, and combinations of these substances. We also considered conditions listed under ICD-10 codes F11-F16, F18, F19, and F55 (excluding nicotine and alcohol, which were noted separately earlier).

### Cause, mechanism, and severity of burn injuries

Burns were categorized based on their potential causes, distinguishing between incidents that occurred at home, during leisure activities, at work, or as a result of traffic accidents. We also made additional distinctions for burn injuries directly linked to alcohol or nicotine consumption.

Injuries associated with pre-existing psychiatric conditions were included as well. Additionally, burns related to neurological conditions such as cerebral ischemia, hemorrhage, multiple sclerosis, paraplegia, epilepsy, dementia, and medical conditions like syncope were included. Uncommon causes of injury, such as train surfing, lightning strikes, and sauna visits, were classified under the category “extreme.”

Our data analysis incorporated burn injury mechanisms and specific characteristics, along with treatment modalities, complications, discharge information, and hospital-mortality outcomes.

Burn injuries were classified according to their mechanisms, namely scalding, explosions, flame exposure, electrical burns, or chemical burns. Frostbite injuries were included as a separate category.

The burn-specific characteristics analyzed included the ABSI and revised Baux scores; burns exceeding 20% TBSA; burns over 10% TBSA in individuals older than 65 years; burns affecting critical areas such as the face, hands, genitals, and major joints; as well as deep-partial thickness burns (DPTB), full-thickness burns (FTB), circumferential burns, verified inhalation injuries (IHI), and additional trauma (eg, ocular injuries, traumatic brain injuries, or mid-facial fractures).

### Polytrauma, diagnosis, and severity classification of TBI

Polytrauma was defined according to the consensus by Butcher et al..^9^ TBI was diagnosed based on patient history, clinical examination using the Glasgow Coma Scale (GCS), and CT scan. In case of a non-intubated patient, a scoring according to GCS was conducted on admission; otherwise, GCS was documented according to patient history given by the admitting first responder team. Classification of the injury severity was done according to the following: mild TBI = GCS 13 – 15; moderate TBI = GCS 9 – 12; severe TBI = 3 – 8.^10^^,^^11^ Head CT scans were analyzed independently by neurosurgeons and neuroradiologists, and descriptive data were collected regarding the presence of skull fractures, spinal fractures, and intracranial injuries, including intracranial hemorrhage and parenchymal lesions.

### Treatment data

Treatment-specific data included the number of surgeries during the ICU stay, mean duration of surgeries, cultured epithelial autografts (CEAs), and enzymatic debridement with Nexobrid® (MediWound, Yavne, Israel). Furthermore, time from admission to first burn wound excision was collected and categorized as “non-delayed” if the surgery had taken place within 72 h post-admission, or “delayed” if it was over 72 h. Available data regarding resuscitation volume given within the first 24- and 48-h post-trauma event, and the duration of intubation and ventilation (as expressed as number of hours on mechanical ventilation) were analyzed.

### Outcome data

Primary outcomes included the number of surgeries, operating time, delay of therapy/burn excision, and hospitalization duration. The complications assessed, diagnosed, and documented in the discharge letters by the ICU team included wound infections, loss of skin grafts or transplants, respiratory infections, urinary tract infections, bacteremia, sepsis, multi-organ failure, gastroparesis, refeeding syndrome, gastrointestinal bleeding, ileus, renal insufficiency, electrolyte disturbances, rhabdomyolysis, anemia, vasoplegia, thrombosis, embolism, cardiac failure (including arrhythmias), agitation, delirium, post-traumatic stress disorder, and post-traumatic depression.

Discharge destinations recorded were home, rehabilitation centers, and other healthcare institutions, such as specialized home-based burn care units for repatriation, regional treatment centers, nursing homes, and psychiatric facilities.

Additionally, length of hospitalization and in-hospital mortality were included as outcomes in the analyses.

### Statistical analyses

Descriptive statistics were summarized using frequency distribution tables. The primary outcomes were analyzed as count data using GLMs with a negative binomial distribution and log link to address data overdispersion. Incidence rate ratios (IRR) were calculated for risk potentiating patient factors (age, psychiatric conditions, substance use, alcohol-related injuries, employment), injury severity (ABSI score, TBSA >20%, critical body regions affected, IHI), treatment details (multiple surgeries, CEAs, Nexobrid®), complications (≥3 events, wound infections), and rehabilitation admissions.

Survival was analyzed as a categorical variable using logistic regression, and results are presented as odds ratios (OR) with corresponding 95% CIs. Predictors included in the analyses were patient demographics, injury characteristics, recurrent surgeries, occurrence of complications, and admission to rehabilitation facilities.

Analyses were conducted using Jamovi (v2.3) and GraphPad Prism (v9.5.1). Continuous variables are shown as mean ± SD, patient age as median (IQR), and categorical variables as percentages. Normality was tested using D’Agostino-Pearson and Shapiro–Wilk tests. Non-normally distributed data underwent Kruskal-Wallis testing with Dunn’s post-hoc analyses. Statistical significance was set at *P* < .05.

## RESULTS

### Patient demographics and comorbidities

Among 27 TBI-burn patients, representing 4.5% of 602 total burned patients, most were male (88.9%), similar to the other groups: burns alone (77.8%), burns with other trauma (88.9%), and electrical burns (95.2%). The median age was 33 years (IQR 16-61), which was consistent with electrical burns patients (33 years) and burns alone (31 years), and burns with other trauma representing the oldest cohort (40 years). Pre-existing psychiatric conditions occurred in 33.3% of TBI-burn patients, which was in line with the range of 23.8%-40.7% among the 3 other cohorts. Alcohol and controlled substance use were each noted in 22.2% of the TBI-burn group, comparable to other cohorts. Detailed results are summarized in Table 1.

**Figure 1:**
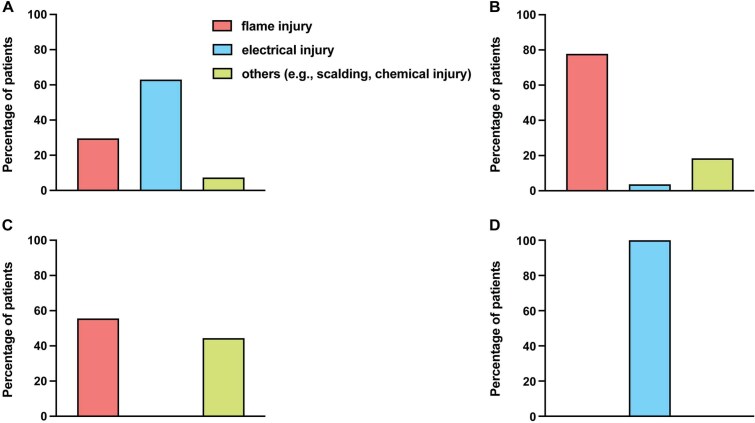
Burn-Injury-Specific Mechanisms. Descriptive count data of binary variables relating to mechanisms for burn injuries are presented for each group: (A) Traumatic brain injury (TBI)-burns: 29.6% flame injuries, 63.0% electric burns, and 7.4% other causes (eg, scalds, chemical burns). (B) Burns alone: 77.8% flame injuries, 3.7% electric burns, and 18.5% other causes. (C) Burns with other trauma: 55.6% flame injuries and 44.4% other causes. (D) Electrical burns: 100.0% electrical injury. Data = counts and %.

**Table 1:** Baseline Demographic Data. Continuous Variables ± IQR, Binary Variables Respectively, Frequency Tables

**Baseline demographic data**	**TBI** ^**a**^ **-burns** **(*n* = 27)**	**Burns alone** **(*n* = 27)**	**Burns with other trauma (*n* = 27)**	**Electrical burns** **(*n* = 21)**
Patient characteristics	**Percentage [%], or median [IQR]**			
Gender				
Male	88.9	77.8	88.9	95.2
Female	11.1	22.2	11.1	4.80
Age [years]	33.0, 16.0% and 61.0% [median, IQR]	31.0, 14.1% and 69.0% [median, IQR]	40.0, 16.8% and 68.0% [median, IQR]	33.0, 16.0% and 67.0% [median, IQR]
16-39	66.7	77.8	44.4	57.1
40-64	29.6	18.5	37.0	33.3
≥65	7.4	3.7	14.8	9.5
Nicotine consumption	22.2	22.2	22.2	9.5
Alcohol consumption	22.2	29.6	40.7	19.0
Psychiatric condition: pre-existing	33.3	40.7	29.6	23.8
Previous suicide attempts	11.1	18.5	7.4	4.8
Controlled substances	22.2	25.9	22.2	0.0
Employed	74.1	66.7	70.4	90.5
Retired	11.1	7.4	3.7	4.8

a TBI = traumatic brain injury.

### Cause, mechanism, and severity of burn injuries

Burns in the TBI cohort primarily resulted from electrical injuries (63%), which is markedly different from the groups burns alone (3.7%) and additional trauma groups (0%), but similar to the electrical burns group (100%). Flame burns occurred in 29.6% of TBI-burn cases versus 77.8% in the burns alone and 55.6% in the additional trauma groups. Burn-injury-specific mechanisms are summarized in Figure 1. Mean ABSI scores were similar across groups: TBI 6.8 (SD ± 2.32), burns alone 7.56 (SD ± 3.45), additional trauma 7.33 (SD ± 2.50), and electrical trauma 6.43 (SD ± 2.93). Burns exceeding 20% TBSA occurred in 66.7% of TBI-burn patients, lower than burns alone (74.1%) and additional trauma patients (88.9%), but higher than electrical trauma patients (52.4%). DPTB and FTB were frequent across all groups: 85.2% in TBI-burn patients, 88.9% in burns alone, 92.6% in burns with other trauma, and 76.2% in electrical burns. Details are summarized in Tables 2 and 3.

**Table 2:** Injury-Specific Characteristic Based on the Potential Causes of Burns. Binary variables, frequency tables

**Group or cause-specific characteristics**	**TBI** ^**a**^ **-burns** **(*n* = 27)**	**Burns alone** **(*n* = 27)**	**Burns with other trauma (*n* = 27)**	**Electrical burns** **(*n* = 21)**
	**Percentage [%]**			
Any home or leisure injuries	48.1	44.4	51.9	0.0
Nicotine-consumption-related	14.8	11.1	3.7	0.0
Current-psychiatric-condition-related	18.5	25.9	22.2	23.8
Injury related to suicide attempts	11.1	22.2	7.4	13.3
Work-related burns	29.6	22.2	29.6	90.5
Traffic-associated burns	18.5	0.0	3.7	0.0
Train surfing	29.6	0.0	0.0	4.8

a TBI = traumatic brain injury.

**Table 3:** Burn-Specific Characteristics. Continuous variables ± SD, binary variables, respectively, frequency tables

**Burn-specific characteristics**	**TBI** ^**a**^ **-burns** **(*n* = 27)**	**Burns alone** **(*n* = 27)**	**Burns with other trauma (*n* = 27)**	**Electrical burns** **(*n* = 21)**
	**Percentage [%] or Mean [SD]**			
Mechanism of injury Flame Electric burn Others (eg, scalding, chemical burns)	29.6 63.0 7.4	77.8 3.7 18.5	55.6 0.0 44.4	0.0 100 0.0
ABSI^b^ score	6.81 (2.32)	7.56 (3.45)	7.33 (2.5)	6.43 (2.93)
Baux score	67.6 (21.9)	68.0 (29.1)	74.9 (27.1)	66.5 (28.2)
>20% TBSA^c^	66.7	74.1	88.9	52.4
Burns of the face, hands, genitals, and larger joints	77.8	7.4	81.5	76.2
Deep-partial and full-thickness burns	85.2	88.9	92.6	76.2
Circular burns	55.6	51.9	51.9	33.3
Verified IHI^d^	29.6	22.2	51.9	14.3
TBI^a^ and spine fractures	18.5	0.0	0.0	0.0
TBI^a^ and ICB^e^	25.92	0.0	0.0	0.0

a TBI = traumatic brain injury,

b ABSI = Abbreviated Burn Severity Index,

c TBSA = total body surface,

d IHI = inhalation injury,

e ICB = intracerebral bleeding.

### Polytrauma, diagnosis, and severity classification of TBI

Polytrauma was present in 9 TBI-burns, 12 in burns combined with other trauma, 1 in electrical burns.

TBI-burn patients had an initial mean GCS of 13.1 (SD ± 3.0). Twenty-two patients were classified as mild, 2 as moderate, and 3 as severe TBI. Further documented injuries included 5 cases of spine fractures (18.5%) and 7 cases of intracranial bleeding (ICB; 25.9%).

### Treatment data

Delayed initial burn wound excision occurred in 6 TBI-burn cases (22.2%). Delay was due to evolving intracranial hemorrhage (*n* = 1), polytrauma and trauma surgeries (*n* = 1), hemodynamic instability due to severe shock (*n* = 3), and need for ICP monitoring (*n* = 1). This delay significantly correlated with age (*P* = .028), burns of the face, hands, genitals, and larger joints (*P* = .045), increased total surgeries (IRR 0.567, *P* = .018), and longer operating times (IRR 1.336, *P* = .048). None of the patients within the other 3 cohorts experienced any delay in burn surgery.

Mean number of plastic surgeries was 6.15 compared to 4.85 for burns alone, 4.22 for burns with other trauma, and 4.19 for electrical burns. Neurosurgical interventions in TBI patients were rare, totaling 6 interventions across 3 patients, consisting of 3 ICP probe implantations, 1 extra ventricular drain (EVD) implantation, 1 change of EVD, and 1 craniectomy and hematoma evacuation. Further surgical interventions included 9 trauma surgeries in 5 patients. Fluid resuscitation in 16 TBI-burn patients was higher within 24 and 48 h (17 204 mL ± 19 155, and18 420 mL ± 12 962, respectively) compared to the other groups: 18 burns alone (10 261 mL ± 6719, and 13 891 mL ± 8701, respectively), 27 additional trauma (14 082 mL ± 9579, and 17 678 mL ± 11 174, respectively), and 16 electrical burns (10 245 mL ± 8769, and 14 262 mL ± 12 770, respectively). No data was available for 11 TBI-burns, 9 burns alone, and 5 electrical burns patients.

Detailed data can be found in Table 4.

**Table 4:** Treatment Modalities, Complications, Hospitalization, Discharge, and Mortality-Data. Continuous variables are presented as mean ± SD; binary variables are shown as frequencies. Missing data: crystalloid fluid therapy within 24 h (TBI^a^-burns: 10, burns alone: 8, electrical burns: 5), and within 48 h (TBI^a^-burns: 11, burns alone: 9, electrical burns: 5); ventilation and intubation duration during hospitalization (TBI^a^-burns: 10, burns alone: 7, Burns with other trauma: 1, electrical burns: 4)

**Therapy-specific characteristics**	**TBI** ^**a**^ **-burns** **(*n* = 27)**	**Burns alone** **(*n* = 27)**	**Burns with other trauma (*n* = 27)**	**Electrical burns** **(*n* = 21)**
	**Volume [mL]** **Percentage [%] or Mean [SD]** **Time [min or h] or Mean [SD]**			
Crystalloid fluid therapy [mL]				
Within 24 h	17 204 (19155)	10 261 (6719)	14 082 (9579)	10 245 (8769)
Within 48 h	18 420 (12962)	13 891 (8701)	17 678 (11174)	14 262 (12770)
Ventilation, intubation during hospitalization [h]	162.0 (195.0)	189.0 (272.0)	288.0 (334.0)	109.0 (143.0)
Number of surgeries	6.15 (5.1)	4.85 (4.63)	4.22 (3.8)	4.19 (4.37)
Plastic	5.22 (4.25)	4.85 (4.63)	4.19 (3.82)	4.05 (4.01)
Neurosurgeries	0.22 (0.7)	0.0 (0.0)	0.0 (0.0)	0.0 (0.0)
Orthopedic	0.33 (0.78)	0.0 (0.0)	0.0 (0.0)	0.14 (0.48)
Duration of surgery [min]	153.0 (76.8)	148.0 (69.6)	164.0 (53.8)	131.0 (74.9)
Plastic	140.0 (58.2)	148.0 (69.6)	164.0 (53.8)	133.0 (74.2)
Neurosurgeries	6.96 (22.5)	0.0 (0.0)	0.0 (0.0)	0.0 (0.0)
Orthopedic	21.8 (56.0)	0.0 (0.0)	0.0 (0.0)	0.48 (2.18)
CEA^b^	14.8	22.2	18.5	9.5
Nexobrid	14.8	37.0	37.0	23.8
Complications	4.04 (4.46)	3.56 (2.86)	3.89 (3.41)	3.43 (3.98)
Wound infections	48.1	29.6	29.6	28.6
Loss of transplant	22.2	7.4	7.4	9.5
Post-traumatic depression	7.4	14.8	40.7	23.8
Hospitalization duration	34.9 (35.1)	45.7 (56.3)	60.2 (89.5)	32.1 (39.6)
Discharge at home	22.2	22.2	18.5	33.3
Rehabilitation	44.4	48.1	40.4	57.1
Discharge, others	14.8	18.5	7.4	4.8
In-hospital mortality	22.2	3.7	7.4	4.8

a TBI = traumatic brain injury,

b CEA = cultured epidermal autograft.

### Outcome data

TBI-burn patients experienced a higher frequency of complications (mean 4.04 complications per patient), notably wound infections (48.1%) compared to 29.6% in burns alone and burns combined with other trauma, and 28.6% in electrical burn patients. Graft loss occurred at a rate of 22.2%, which was higher than burns alone and burns combined with other trauma (both 7.4%) and electrical burn patients (9.5%). Median ventilation and intubation time was 162 h (*n* = 17 patients), which was not longer than the other groups. However, burns combined with other trauma had significantly extended ventilation and intubation time compared to electrical burns (*P* = .026). Hospital stays averaged 34.9 days, which was within the range of the other 3 cohorts (32.1-60.2 days). Mortality was higher in patients with TBI (22.2%) compared to burns alone (3.7%), burns combined with other trauma (7.4%), and electrical burns (4.8%). A significant interaction was found between delayed therapy due to TBI and increased hospitalization durations for burns involving critical areas (IRR 8.631, *P* = .002). Lastly, patients with TBI were significantly more prone to suffer from a delay in burn excision when increased age (*P* = .028) or burns of the face, hands, genitals, and larger joints (*P* = .045) were present. Detailed data is summarized in Tables 5, 6, 7, and 8.

**Table 5:** Regression Results: Number of Surgeries and Different Risk Factors. Positive significant correlation between the number of surgeries and age, past medical history of alcohol consumption, burns of the face, hands, genitals, and larger joints, deep-partial and full-thickness burns, delay in therapy due to TBI^a^, and complication rate ≥2, respectively. Generalized linear model (GLM) with negative binomial distribution and a log link due to a skewed distribution. Significant interactions between burns of the face, hands, genitals, and larger joints and complication rate ≥2

**Outcome: number of surgeries (counts), GLM with negative binomial distribution**		
	**IRR** ^**b**^ **(95% CI**^**c**^**)**	***P*-value**
**Age**	**0.966 (0.945-0.987)**	**.002**
**Alcohol consumption**	**1.913 (1.207-3.016)**	**.005**
**Unemployment status**	0.451 (0.107-1.303)	.197
**Burns of the face, hands, genitals, and larger joints**	**2.204 (1.223-3.940)**	**.008**
**Deep-partial and full-thickness burns**	**0.462 (0.232-0.880)**	**.022**
**ABSI** ^**d**^ **score**	1.079 (0.985-1.182)	.101
**Delay in therapy due to TBI^**a**^**	**0.567 (0.352-0.901)**	**.018**
**Complications ≥2**	**2.637 (1.499-4.615)**	**<.001**
**Burns associated to train surfing**	0.985 (0.587-1.647)	.955
Burns of the face, hands, genitals, and larger joints* Complications ≥2:	**5.868 (1.765-19.179)**	**.004**

a TBI = traumatic brain injury,

b IRR = incidence rate ratio,

c CI = confidence interval,

d ABSI = Abbreviated Burn Severity Index.

**Table 6:** Regression Results: Operating Time and Different Risk Factors. Positive significant associations between operating time and burns of the face, hands, genitals, and larger joints, delay in therapy due to TBI^a^, and number of surgeries ≥2, respectively. Generalized linear model (GLM) regression model with negative binomial distribution and a log link due to a skewed distribution. Significant interactions between deep-partial and full-thickness burns and number of surgeries ≥2

Outco**me: operating time (counts), GLM with negative binomial distribution**		
	**IRR** ^**b**^ **(95% CI**^**c**^**)**	***P*-value**
**Age**	1.000 (0.990-1.009)	.935
**Alcohol consumption**	0.812 (0.608-1.089)	.176
**Unemployment status**	1.449 (0.852-2.592)	.184
**Burns of the face, hands, genitals, and larger joints**	**0.564 (0.415-0.770)**	**<.001**
**Deep-partial and full-thickness burns**	0.796 (0.509-1.254)	.326
**ABSI** ^**d**^ **score**	1.045 (0.981-1.113)	.161
**Delay in therapy due to TBI^**a**^**	**1.336 (1.004-1.779)**	**.048**
**Number of surgeries ≥2**	**0.536 (0.369-0.789)**	**.001**
**Burns associated to Train surfing**	147.273 (103.332-214.026)	.775
Deep-partial and full-thickness burns * Number of surgeries ≥2:	**0.122 (0.055-0.276)**	**<.001**

a TBI = traumatic brain injury,

b IRR = incidence rate ratio,

c CI = confidence interval,

d ABSI = Abbreviated Burn Severity Index.

**Table 7:** Regression Results: Hospitalization with Different Risk Factors. A positive association was found between hospitalization duration and unemployment status. Generalized linear model (GLM) with negative binomial distribution and a log link due to a skewed distribution. Significant interactions between delay in therapy due to TBI^a^ and burns of the face, hands, genitals, and larger joints

**Outcome: hospitalization duration (days, counts), GLM with negative binomial distribution**		
	**IRR** ^**b**^ **(95%-CI**^**c**^**)**	***P*-value**
**Age**	0.980 (0.959-1.000)	.064
**Alcohol consumption**	1.067 (0.526-2.222)	.850
**Unemployment status**	**0.079 (0.016-0.384)**	**<.001**
**Burns of the face, hands, genitals, and larger joints**	1.741 (0.866-3.606)	.117
**Deep-partial and full-thickness burns**	0.438 (0.187-1.062)	.054
**ABSI** ^**d**^ **score**	1.098 (0.941-1.287)	.140
**Delay in therapy due to TBI** ^**a**^	0.892 (0.476-1.698)	.726
**Burns associated to Train surfing**	1.618 (0.689-3.845)	.204
**Delay in therapy due to TBI** ^**a**^ * Burns of the face, hands, genitals, and larger joints	**8.631 (2.149-36.226)**	**.002**

a TBI = traumatic brain injury,

b IRR = incidence rate ratio,

c CI = confidence interval,

d ABSI = Abbreviated Burn Severity Index.

**Table 8:** Regression Results: Delay in Therapy/Excision Due to TBI and Different Risk Factors. Positive correlations were found between delay in therapy due to TBI and burns of the face, hands, genitals, and larger joints and age. Logistic regression model for binary data. Estimates represent the log odds of “survival = yes” vs “survival = no”. No significant interactions between the variables

**Outcome: delay in therapy/excision due to TBI** ^**a**^ **(%), logistic regression model**		
	**Odds ratio (95% CI** ^**b**^ **)**	***P*-value**
**Age**	**0.687 (0.49-0.961)**	**.028**
**Alcohol consumption**	335.809 (0.270-417019.543)	.110
**Burns of the face, hands, genitals, and larger joints**	**9399.495 (1.238-7.13e+7)**	**.045**
**Deep-partial and full-thickness burns**	5.981 (0.022-1664.333)	.533
**ABSI score**	3.072 (0.779-12.115)	.109
**Verified IHT** ^**c**^	28.155 (0.200-3956.365)	.533
**Burns associated to train surfing**	0.080 (1.76e-4-1.09e-8)	2.843

a TBI = traumatic brain injury,

b CI = confidence interval,

c IHT = inhalation injury.

## DISCUSSION

Concomitant TBI profoundly complicates clinical management and negatively impacts outcomes in burn patients. Although relatively uncommon, affecting only 4.5% (27 of 602) of burned patients at our specialized tertiary burn and neurotrauma center, the presence of TBI markedly increased mortality and morbidity compared to isolated burn injuries. Despite the robust demographic profile, with primarily young adults, patients with concomitant TBI showed substantially worse clinical trajectories. This observation aligns with existing literature indicating poorer outcomes among burn patients experiencing neurological injury.

Our TBI-burn cohort consisted primarily of young adult males (88.9%) with a median age of 33 years, and notably one-third having some form of pre-existing psychiatric condition. This demographic profile is important, as psychiatric comorbidities and young age can influence risk behaviors and recovery, but even within this relatively robust patient group, the outcomes were markedly worse when TBI accompanied the burn injury. This is in line with our recent study about the impact of mental health in ICU burn patients.^12^ While neurosurgical interventions were rare, with only 3 patients requiring cranial surgeries, mortality in TBI-burn patients (22.2%) was considerably higher than in the other groups (3.7%-7.4%), although the difference did not reach statistical significance due to sample size limitations. Still, the greater mortality in the TBI-burn group relative to burns accompanied by non-neurological polytrauma may be due to a disproportionate impact of neurologic injury compared to polytrauma without neurologic injury. However, it is important to point out that 63% of our TBI-burn cohort had electrical burns, which are typically associated with more severe tissue damage and multiple additional traumas. This factor likely contributed to the observed high mortality rate and is likely to not only be due to TBI. Nevertheless, our findings align with previous literature reporting significantly higher mortality in burn patients with concomitant TBI compared to those without head injuries.^5^ These findings underscore the poor prognosis and emphasize the importance of specialized multidisciplinary management for this patient population.

Fluid resuscitation strategies for burn patients with concurrent TBI represent a critical clinical challenge, balancing effective burn shock management against risks of exacerbating cerebral edema. Current burn resuscitation protocols, such as the Parkland formula, advocate liberal crystalloid volumes, titrated to urine output and potentially supplemented with albumin or plasma.^13^ However, this approach may conflict with neuroprotective strategies emphasizing minimal fluid overload and avoidance of hypo- or hypernatremia.^14–16^ While our data did not demonstrate significantly different fluid volumes administered in the initial 24 and 48 h using the Parkland formula across groups, individualized fluid management remains essential, with hypertonic saline solutions potentially bridging burn and neuroprotective resuscitation needs.^17–20^

Severe TBI triggers a cascade of neuroinflammatory and neurohormonal changes that can disrupt the blood-brain barrier (BBB), precipitate cerebral edema,^21^^,^^22^ and exacerbate the hypermetabolic and immunosuppressive state associated with major burns. Subsequent increase in systemic inflammation and immune dysregulation following burn injury further contribute to BBB disruption, glial activation, and neuronal damage, compounding the primary injury caused by TBI.^21–23^ Burn patients frequently develop metabolic encephalopathies, such as acute hyponatremia due to fluid shifts, which may provoke confusion or seizures, potentially leading to osmotic demyelination if corrected too rapidly, or hypernatremia resulting from evaporative fluid losses,.^23^ These mechanisms suggest that a burn injury can exacerbate TBI’s secondary brain injury cascade through systemic inflammation and hemodynamic instability. The critical care environment itself, involving sedation, mechanical ventilation, and invasive monitoring, may obscure neurologic signs and complicate timely interventions.

Furthermore, systemic immune suppression related to TBI significantly increases infection risk.^22^^,^^24^^,^^25^ Major burn patients are inherently susceptible to infection due to compromised skin barriers and burn-induced immune dysfunction, and the presence of concomitant TBI appears to further exacerbate this vulnerability. In our cohort, wound complications were notably frequent among TBI patients, with nearly half (48.1%) experiencing wound infections; however, this increase compared to approximately 30% in other cohorts did not reach statistical significance. Additionally, graft loss was common, affecting over one-fifth of TBI-burn patients, suggesting impaired wound healing. Consistent with our observations, military case series have demonstrated significantly higher rates of sepsis, pneumonia, wound infections, and urinary tract infections in burn patients with concurrent TBI compared to those without neurological injury.^24^^,^^26^

Prolonged intubation and critical care for TBI augment the typical burn-related infection risks. Neurocritical care often involves invasive procedures, such as ICP monitoring, mechanical ventilation, and urinary catheterization, all of which provide potential entry points for pathogens in an already immunocompromised host. Specifically, hydrotherapy—an essential component of burn wound management—was restricted in one patient at our institution due to contamination risks associated with an ICP probe. Additionally, the ICP probe prevented to harvest of split-thickness skin grafts from the scalp, further complicating wound management.

Burn-induced impairment of both innate and adaptive immunity leads to opportunistic infections, occasionally involving the central nervous system.^27^ For instance, burn patients with severe sepsis are known to occasionally develop infectious meningitis, most often from *Pseudomonas aeruginosa* or other hospital-acquired bacteria translocating from burn wounds or lung infections.^22^^,^^27^ This rare complication underscores how burns can predispose to unusual neurologic infections. Therefore, infection prevention is paramount: strict aseptic technique in wound care, early excision of necrotic tissue, and appropriate antibiotic use are critical in burn patients, especially when a TBI is present and could be exacerbated by any septic encephalopathy.

Early excision and grafting are the cornerstones of burn care; however, the presence of a TBI often necessitates delays or staging of surgical interventions. The timing of burn wound excision and grafting in patients with TBI must balance the benefits of early wound closure against the risks of operative stress on a neurologically injured patient. Early excision of deep burns within the first 24-48 h is standard of care in burn surgery as it reduces infection risk, promotes wound healing, and improves survival compared to delayed excision.^28^ Seventy-two hours seems to be acceptable for certain patient groups as well.^29^ Consequently, unnecessary delays in burn wound excision should be avoided, as prolonged exposure of wounds significantly increases the risk of infectious and inflammatory complications, potentially exacerbating neurologic injury. In our study, such delays correlated with a more complex surgical course. Patients in the TBI cohort required significantly more operative interventions and spent longer cumulative time in surgery to achieve adequate wound coverage, reflecting the need for multiple staged debridements and grafting procedures, even when matched with and adjusted for burn severity compared to the other 3 groups. Each postponement likely facilitated increased bacterial colonization, contributing to higher observed rates of infection and graft loss. Optimal management of these complex cases mandates close interdisciplinary collaboration involving burn specialists, neurosurgeons, anesthesiologists, intensivists, and infectious disease specialists. Intraoperative strategies to mitigate increases in ICP, including adequate anesthesia, mild hyperventilation, and head elevation, are routinely implemented. In patients experiencing severe TBI with refractory intracranial hypertension, it may be necessary to stage surgical excisions or adopt interim measures, such as escharotomies and antimicrobial dressings, until the neurological status allows more invasive surgery. Nevertheless, early tangential excision and grafting, combined with vigilant perioperative neuromonitoring, likely represents the best strategy to minimize the septic and metabolic complications in these patients. Therefore, a balanced approach that simultaneously addresses critical neurotrauma management and burn-related sepsis is essential to optimize clinical outcomes.

Our study has several limitations, including its retrospective, single-center design, the low incidence of concomitant TBI, and incomplete data on fluid resuscitation and ventilation duration, potentially restricting generalizability. Future prospective multicenter investigations are necessary to validate our findings and refine clinical guidelines further. Despite these limitations, our results emphasize the necessity of multidisciplinary, tailored clinical pathways in managing combined burn and TBI injuries, aiming to optimize outcomes in this challenging patient population.

## CONCLUSIONS

Taken together, our study underscores that, despite their rarity, patients with burns complicated by TBI constitute a uniquely vulnerable cohort characterized by high morbidity and mortality. The pathophysiological interactions between TBI and burn injuries increase infection rates, graft failures, and mortality risk, resulting in prolonged hospitalization and a greater demand for surgical interventions and intensive supportive care. Therefore, a tailored, multidisciplinary management strategy is essential, prioritizing timely surgical interventions while preserving neuroprotective measures. Ongoing research and the establishment of specialized treatment protocols are crucial to addressing the distinctive clinical challenges posed by this complex patient group and ultimately improving their survival and functional outcomes.

## Data Availability

The datasets used and/or analyzed during the current study are available from the corresponding author on reasonable request.

## Notes

Author contributions: Mauro Vasella (Conceptualization, Data curation, Formal analysis, Investigation, Methodology, Project administrator, Supervision, Visualization, Writing—original draft, Writing—review & editing), Michael-Alexander Pais (Data curation, Formal analysis, Investigation, Methodology, Software, Visualization, Writing—original draft, Writing—review & editing), Lukas Naef (Data curation, Validation, Writing—review & editing), Matthias Haenggi (Methodology, Validation, Writing—review & editing), Giovanna Brandi (Methodology, Validation, Writing—review & editing), Emanuela Keller (Validation,Writing—review & editing), Victor E. Staartjes (Investigation, Validation, Writing—review & editing), Luca Regli (Validation, Writing—review & editing), Pietro Giovanoli (Validation, Writing—review & editing), Bong-Sung Kim (Validation, Writing—original draft), and Flavio Vasella (Conceptualization, Data curation, Formal analysis, Investigation, Methodology, Project administrator, Supervision, Writing—original draft, Writing—review & editing). All authors read and approved the final manuscript.

Funding: No funding was received for this study. Bong-Sung Kim is a consultant of Kerecis®; however, he did not receive any financial support for this work.
